# Research progress and prospects of exosomes from diverse cellular sources in the treatment of knee osteoarthritis: a narrative review

**DOI:** 10.3389/fsurg.2026.1762559

**Published:** 2026-03-06

**Authors:** Xinyu Li, Zhiyong Li, Qi Zhao, Xiaoyang Zhou, Yubo Shi, Sheng Zhou, Peng Duan, Guoxin Huang, Yihua Shi

**Affiliations:** 1 Department of Orthopedics, Xiangyang No.1 People’s Hospital, Hubei University of Medicine, Xiangyang, China; 2 Department of Orthopedics, Postgraduate Union Training Base of Xiangyang No.1 People’s Hospital, School of Medicine, Wuhan University of Science and Technology, Xiangyang, China; 3 Key Laboratory of Zebrafish Modeling and Drug Screening for Human Diseases of Xiangyang City, Xiangyang No. 1 People’s Hospital, Hubei University of Medicine, Xiangyang, China

**Keywords:** exosome, injections, intra-articular, knee osteoarthritis, regenerative medicine

## Abstract

Knee osteoarthritis (KOA) is a highly prevalent degenerative joint disease characterized by osteophyte formation at joint margins, subchondral bone sclerosis, and progressive degeneration of the articular cartilage. In advanced stages, it can result in severe functional impairment of the knee joint, imposing a substantial burden on patients’ quality of life and on healthcare systems. Conventional intra-articular treatments, such as hyaluronic acid and corticosteroid injections, provide temporary pain relief but fail to achieve true tissue repair or regeneration. In recent years, several novel biological agents with regenerative potential have been introduced, yet their efficacy and safety remain debated. Exosomes, nanoscale vesicles secreted by cells, play essential roles in the pathophysiology of OA by mediating intercellular communication and regulating inflammatory and metabolic processes within the joint microenvironment. Exosomes derived from various cellular sources have been shown to promote chondrocyte proliferation and survival, suppress inflammation, maintain cartilage matrix homeostasis, and modulate subchondral bone remodeling and angiogenesis, demonstrating significant therapeutic promise for KOA. This review systematically summarizes current research on the mechanisms and therapeutic potential of exosomes derived from diverse cell types in KOA, highlighting recent advances and ongoing challenges. It aims to provide a theoretical foundation and reference framework for future basic studies and clinical translation of exosome-based therapies.

## 1 Introduction

Knee osteoarthritis (KOA), the most prevalent chronic degenerative joint disease worldwide, is a leading cause of disability and reduced quality of life among middle-aged and older adults (1). Its prevalence shows a marked increase with advancing age (2, 3). The primary clinical manifestations of KOA include knee pain and restricted mobility, while its pathological features involve progressive cartilage wear, degeneration, and osteophyte formation (4, 5). Globally, developed countries report higher prevalence rates due to population aging and improved diagnostic capabilities (6), whereas developing nations face a rapidly growing disease burden driven by lifestyle changes, rising obesity rates, and increased life expectancy (7).

The central pathogenesis of KOA involves abnormal mechanical stress that triggers intra-articular inflammation, leading to excessive protease activation and cartilage matrix degradation that exceeds the tissue's repair capacity (8). This cascade also promotes osteophyte formation and synovitis, ultimately resulting in irreversible progressive joint destruction (9). In clinical practice, patients with advanced-stage KOA and severe joint damage often require surgical intervention when conservative treatments fail. However, in early-stage KOA, pathological damage to tissues such as articular cartilage and subchondral bone is considerably less severe than in intermediate or advanced stages, rendering surgery unnecessary (10). Treatment options at this stage remain limited. With evolving diagnostic and therapeutic paradigms, increasing emphasis has been placed on early detection, precise diagnosis, and stepwise intervention. In this context, recent years have witnessed notable advances in regenerative medicine approaches for KOA.

Intra-articular injection therapy has emerged as a key modality in the regenerative treatment of OA, encompassing a range of therapeutic approaches. Increasing evidence supports its efficacy, particularly within the field of orthobiologics, where research activity has expanded markedly in recent years (11). Established treatment options include hyaluronic acid for lubrication, corticosteroids for anti-inflammatory effects, and stem cells with immunomodulatory and tissue-regenerative potential. These therapies have shown favorable outcomes in reducing inflammation, promoting tissue repair, and improving joint function (12). However, despite their therapeutic promise, these interventions are often limited by challenges such as inconsistent efficacy, lack of standardized preparation protocols, and ethical concerns, all of which hinder their clinical translation and broader application (13, 14). In this context, exosomes—nanoscale extracellular vesicles secreted by cells—have garnered significant attention for their unique biological functions. Carrying bioactive molecules such as proteins, nucleic acids, and lipids, exosomes play critical roles in intercellular communication, immunomodulation, and tissue repair. Moreover, they have demonstrated substantial potential for cartilage protection and regeneration in the treatment of KOA (15).

This review systematically examines the therapeutic potential and developmental challenges of exosomes in the treatment of KOA. It discusses the biological characteristics of exosomes, their mechanisms of action in KOA pathogenesis, recent advances in preclinical studies, and the current bottlenecks in clinical translation, while also outlining future directions. The goal is to provide a comprehensive reference for researchers, clinicians, and scholars in related fields—facilitating the translation of exosome-based therapies from basic research to clinical practice and ultimately offering more effective and minimally invasive treatment options for patients with KOA.

## 2 Pathological mechanisms of KOA

KOA is fundamentally a whole-joint disorder driven by the combined effects of mechanical and biological factors, resulting in impaired integrity of the articular cartilage, subchondral bone, and surrounding tissues, accompanied by inflammatory responses (16). Its pathological mechanism is complex and progressive in nature, typically beginning with the degeneration of articular cartilage (17). Under continuous stimulation from abnormal mechanical loading (e.g., obesity, trauma) and pro-inflammatory cytokines (such as IL-1 and TNF-α), chondrocyte metabolism becomes disrupted, marked by reduced synthetic capacity and heightened catabolic activity (18, 19). Chondrocytes produce excessive amounts of degrading enzymes, including matrix metalloproteinases (MMPs) and aggrecanases, which break down key components of the extracellular matrix (ECM), such as type II collagen and proteoglycans (20). This degradation leads to structural deterioration, loss of elasticity, fissuring, and erosion of the cartilage, ultimately causing cartilage thinning, fragmentation, and loss of joint cushioning (21, 22).

Importantly, the pathology extends beyond the cartilage. Synovial tissue develops secondary inflammation, releasing additional inflammatory mediators and perpetuating a self-sustaining cycle (8). Subchondral bone undergoes sclerosis, cyst formation, and osteophyte growth in an attempt to compensate, which paradoxically increases joint stiffness and pain (23). Surrounding muscles and ligaments also weaken and lose function due to pain and reduced mobility (24). Overall, the pathological process forms a vicious cycle in which mechanical damage and biological responses reinforce one another, culminating in structural failure of the joint, functional impairment, and clinical symptoms such as pain, stiffness, and restricted movement (25).

## 3 Biological characteristics of exosomes

**Figure 1:**
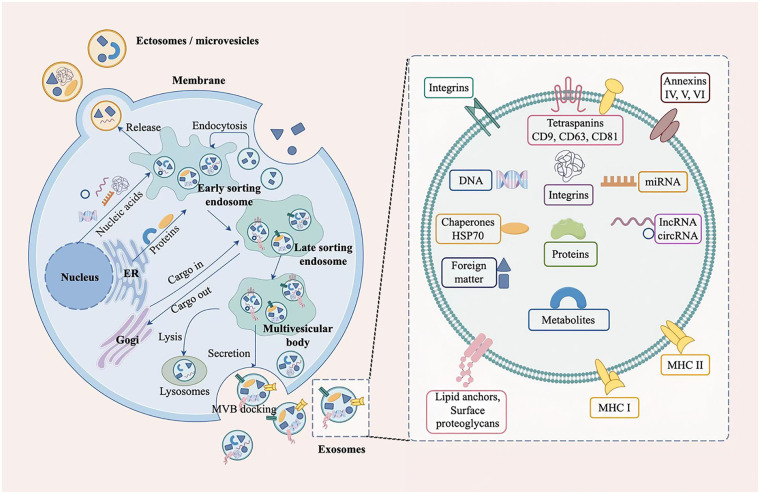
Biogenesis and release of exosomes and structure of exosomes. Exosome formation initiates with the inward invagination of the plasma membrane, which creates an early sorting endosome. This compartment then matures into a late sorting endosome. The membrane of the late endosome subsequently buds inward, accumulating intraluminal vesicles and transforming the structure into a multivesicular body. Finally, the multivesicular body fuses with the plasma membrane. Through exocytosis, it releases its internal vesicles into the extracellular space as exosomes. Exosomes possess a lipid bilayer membrane. This membrane incorporates various transmembrane proteins and receptors. Internally, exosomes carry a diverse cargo of functional biomolecules, including proteins and nucleic acids.

**Figure 2:**
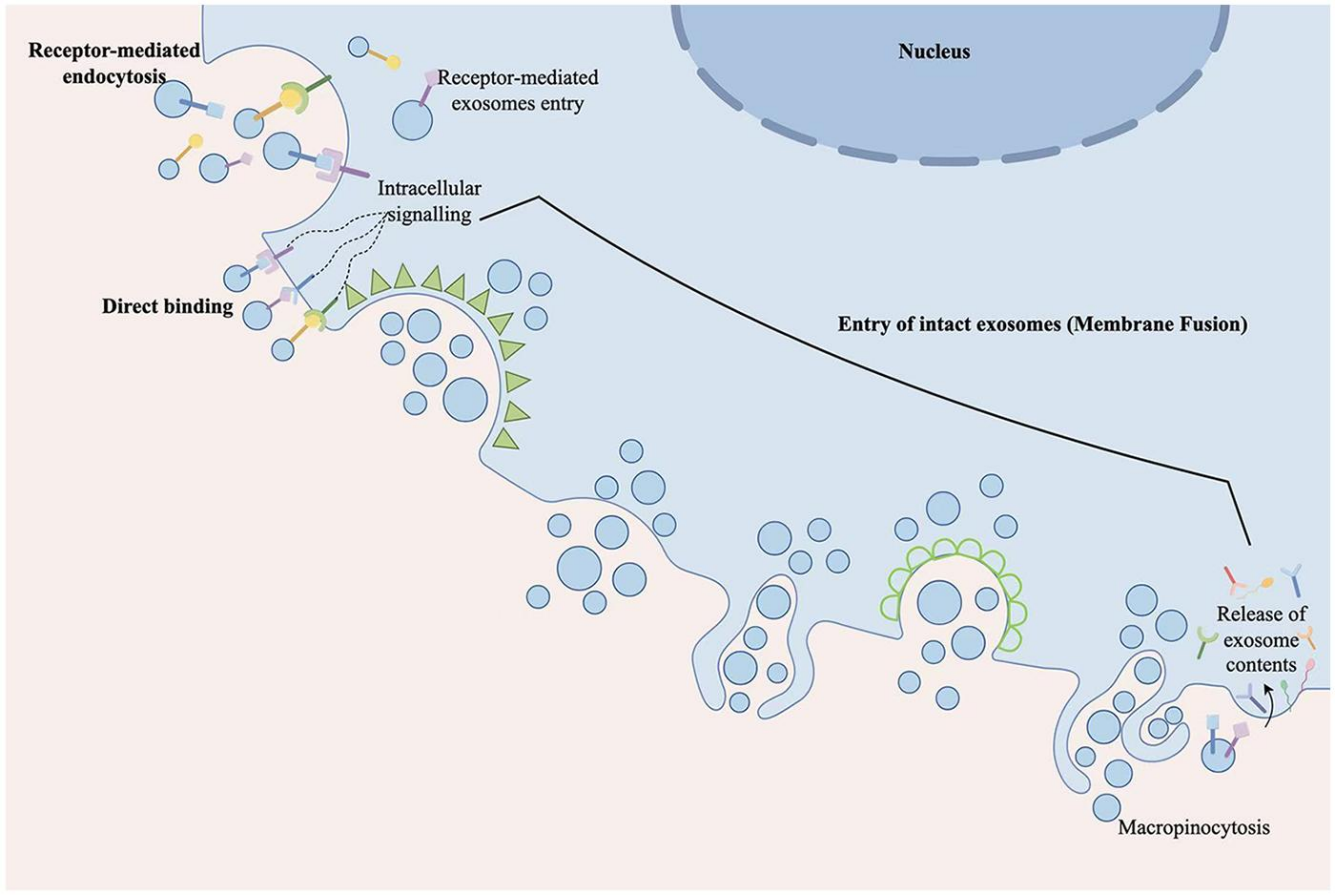
Uptake of exosomes. The release of exosomes mainly occurs through the following three mechanisms: receptor-mediated endocytosis, direct binding, and membrane fusion.

Extracellular vesicles (EVs) are classified into several subtypes based on their size and mechanisms of biogenesis, with exosomes representing one of the principal categories (26), as summarized in Table 1. Exosome biogenesis is a tightly regulated, multi-step process originating from various cell types within tissues (27). This process involves key stages, including the formation of early endosomes, maturation into late endosomes, assembly of multivesicular bodies, and ultimately the release of small vesicles—illustrated in Figure 1. As illustrated in Figure 2, the exosome membrane surface displays structures including receptors and lipid anchors. Internally, exosomal proteins consist primarily of heat shock proteins, tetraspanin family members, and other membrane-associated proteins. Furthermore, exosomes carry a variety of functional nucleic acids, such as mitochondrial DNA, mRNA, circular RNA, microRNA, and long non-coding RNA (28). In addition, exosomes play essential roles in numerous biological processes such as angiogenesis, apoptosis, antigen presentation, intercellular signaling, and inflammatory regulation. Through mediating intercellular communication, they enable precise modulation of recipient cells and act as critical carriers of molecular information between cells (29). Studies have shown that multiple cell types within the joint cavity, including chondrocytes and synovial cells, are capable of secreting exosomes (30). Under physiological conditions, chondrocyte-derived exosomes help regulate the balance between ECM synthesis and degradation by transferring specific miRNAs, thereby maintaining the structural integrity and functional stability of cartilage tissue (31).

**Table 1:** Different types of EVs.

Classification	Type	Size (nm)	Origin	Composition	Key markers
Small EVs	Exomeres (26)	<50	Most cell types	Non-membranous nanoparticles	Non-membranous
	Exosomes (32, 33)	30–150	Most cell types	Proteins, nucleic acids, lipids	Tetraspanins (CD9, CD63, CD81, and CD82), ESCRT family of proteins, heat shock proteins (HSP70, HSP90), Alix, Tsg101
	Microvesicles (26, 33, 34)	>200	Most cell types	Plasma membrane, lipids	Tetraspanins, proteins related to the germination process
Large EVs	Migrasomes (35, 36)	500–3000	Cells in migration	Proteins, mRNAs	Tetraspanins, CPQ, PIGK, NDST1, EOGT
	Apoptotic bodies (37, 38)	1,000–5,000	All cell types	Proteins, RNAs, nuclear components, lipids, gasotransmitters	Integrin alpha-5, calreticulin
	Large oncosomes (39, 40)	1,000–10,000	Cancer cells	Proteins, nucleic acids, lipids	ARF6, metalloproteinases

Endosomal sorting complexes required for transport, CPQ, carboxypeptidase Q; PIGK, phosphatidylinositol glycan anchor biosynthesis, class K; NDST1, bifunctional heparan sulfate N-deacetylase/N-sulfotransferase 1; EOGT, EGF domain–specific O-linked N-acetylglucosamine transferase.

## 4 Exosomes serve as diagnostic biomarkers for osteoarthritis

Early-stage osteoarthritis is frequently asymptomatic, yet treatment becomes increasingly difficult as the condition advances. This reality underscores the necessity of employing biomarkers for early diagnosis. Exosomes directly come from the damaged joint tissue. Their contents can dynamically and precisely reflect the early pathological changes of osteoarthritis, thereby overcoming the limitations of traditional imaging examinations (41).

At present, numerous differentially expressed molecules associated with the onset and progression of osteoarthritis have been identified in exosomes. Among these, microRNAs represent the most extensively studied class of biomarkers. For example, exosomal miR-193b-3p is downregulated in osteoarthritis patients, whereas miR-130b-3p and miR-1271-5p are upregulated (42). Additionally, aberrant expression of the chondroprotective miR-140 is linked to cartilage defects (43). Moreover, long non-coding RNAs (lncRNAs) and circular RNAs (circRNAs) exhibit high stability. This property makes them promising biomarker candidates. For instance, the expression of lncRNAs prostate-specific transcript 1 and PCGEM1 in synovial exosomes rises with disease progression (44). This increase can help distinguish between different disease stages. Meanwhile, plasma circRNA-016901 shows specific overexpression in osteoarthritis patients. Its expression level correlates with disease severity, indicating its potential as a circulating biomarker (45).

For diagnostic targeting, synovial fluid exosomes represent the most direct and sensitive source for detecting early osteoarthritis, as they closely mirror the initial pathological and physiological alterations within the joint. Hence, they are often regarded as the reference standard for early diagnosis. In contrast, blood-derived exosomes, despite some signal dilution, offer the advantages of being minimally invasive and easily obtainable over time. These properties make them a primary candidate for clinical translation and longitudinal disease monitoring. In summary, exosomes carry specific molecular profiles, including miRNAs, lncRNAs, circRNAs, and proteins. This cargo provides a novel and multidimensional platform for molecular analysis. This platform can be applied to the early diagnosis of osteoarthritis, disease staging, and progression prediction.

## 5 Application of exosomes from different sources in the treatment of KOA

### 5.1 Bone marrow mesenchymal stem cell–derived exosomes (BMSC-Exos)

BMSCs are a population of multipotent cells residing in the bone marrow that play crucial roles in tissue repair and immunomodulation. The exosomes secreted by these cells are nanoscale vesicular structures containing a variety of bioactive molecules, including proteins and nucleic acids (46). BMSC-Exos facilitate intercellular communication and regulate the functions of neighboring cells. Studies have shown that BMSC-Exos effectively promote tissue regeneration and repair damaged cartilage and subchondral bone (47). Moreover, they exhibit low immunogenicity and favorable safety profiles in therapeutic use, making them a promising strategy for the treatment of KOA (48).

Existing studies have shown that BMSC-Exos enhance cartilage matrix synthesis by upregulating the expression of type II collagen and aggrecan, while simultaneously inhibiting catabolic enzymes such as ADAMTS5 and MMP-13 to slow matrix degradation (49). They also modulate key signaling pathways through the delivery of regulatory RNAs. For example, miR-361-5p targets DDX20, leading to inactivation of the NF-*κ*B signaling pathway and attenuation of inflammatory responses (50); lncRNA NEAT1 binds to miR-122-5p, preventing it from targeting Sesn2 and thereby activating the Nrf2 pathway to inhibit chondrocyte senescence and apoptosis (51); and miR-127-3p reduces MMP-13–mediated type II collagen degradation by suppressing the Wnt/*β*-catenin signaling pathway (52), as summarized in Table 2. Collectively, these studies demonstrate that BMSC-Exos regulate intra-articular inflammatory responses, promote chondrocyte regeneration, and inhibit ECM degradation by transferring various microRNAs and lncRNAs. Through these mechanisms, they play a crucial role in maintaining cartilage microenvironment homeostasis and delaying the pathological progression of KOA.

**Table 2:** Mechanisms of action and therapeutic effects of BMSC-exos in the treatment of OA.

RNA	Mechanism	Effect	Reference
miR-361-5p	Targets DDX20 and inactivates the NF-*κ*B signaling pathway	Alleviates inflammation and chondrocyte damage	(50)
miR-362-5p	Targets PLXNB1	Promotes chondrogenic differentiation	(53)
miR-127-3p	Targets CDH11 and activates the Wnt/*β*-catenin pathway	Alleviates chondrocyte damage	(52)
miR-21	Upregulates circYAP1 gene while downregulating TLR7 gene	Alleviates inflammation and oxidative stress	(54)
miR-9-5p	Inhibits SDC1	Alleviates inflammation and chondrocyte damage	(55)
miR-320c	Targets CDK6 gene, inhibits IL-1β, and regulates the NF-κB signaling pathway	Inhibits chondrocyte catabolism	(56)
miR-92a-3p	Regulates ADAMTS-4/5 factors	Alleviates inflammation while maintaining cartilage developmental homeostasis	(57)
miR-125a-5p	Targets E2F2	Promotes chondrocyte migration and inhibits cartilage degeneration	(58)
miR-136-5p	Targets ELF3	Promotes chondrocyte migration and inhibits cartilage degeneration	(59)
lnc RNA NEAT1	Binds to miR-122-5p and activates the Sesn2/Nrf2 axis	Induces chondrocyte proliferation and autophagy while inhibiting apoptosis	(51)

### 5.2 Adipose mesenchymal stem cell–derived exosomes (ADSC-Exos)

ADSCs are adult stem cells isolated from adipose tissue, possessing notable regenerative and reparative potential (60). They are capable not only of differentiating into multiple cell types but also of modulating immune responses, promoting angiogenesis, and facilitating tissue repair through the secretion of diverse bioactive factors (61). ADSC-Exos, the nanoscale vesicles released by ADSCs, function as crucial signaling mediators that deliver targeted repair signals to injured cells (62). Given the wide distribution and abundance of adipose tissue in the human body, ADSCs offer significant advantages such as ample availability and ease of collection. As a result, ADSC-Exos are considered a safer and more accessible therapeutic option for KOA, showing great promise in the field of regenerative medicine (63).

ADSC-Exos alleviate intra-articular chondrocyte inflammation by suppressing the release of inflammatory mediators (64). One study demonstrated that miR-338-3p derived from ADSCs inhibits IL-1β–induced chondrocyte inflammation and matrix degradation by downregulating RUNX2 expression (65). In addition, ADSC-Exos can mitigate KOA through multiple signaling pathways. Both *in vivo* and *in vitro* studies have shown that ADSC-derived exosomes deliver the key functional molecule miR-376c-3p to articular chondrocytes and synovial fibroblasts, directly inhibiting the protein expression of WNT3 and WNT9a. This, in turn, reduces activation of the downstream signaling molecule *β*-catenin, leading to marked suppression of ECM degradation in chondrocytes and alleviation of synovial fibrosis (66). Furthermore, recent studies have found that under inflammatory conditions mimicking KOA *in vitro* (induced by IL-1β), the expression of miR-574-3p in ADSC-derived exosomes is significantly downregulated. Mechanistic analyses revealed that miR-574-3p directly targets CRIM1 through molecular binding, thereby inhibiting its protein translation and reducing CRIM1 expression. This effect subsequently relieves the suppression of BMP signaling, resulting in dual therapeutic benefits: inhibition of chondrocyte hypertrophy and attenuation of inflammatory responses (67). Details are presented in Table 3.

**Table 3:** Mechanisms of action and therapeutic effects of ADSC-exos in the treatment of OA.

RNA	Mechanism	Effect	Reference
miR-338-3p	Inhibits RUNX2	Inhibits chondrocyte inflammation and degradation	(65)
miR-376c-3p	Inhibits Wnt/β-catenin signaling pathway by targeting WNT3 or WNT9a	Alleviates chondrocyte degradation and synovial fibrosis	(66)
miR-574-3p	Modulates CRIM1/BMPs signaling	Inhibits chondrocyte hypertrophy and inflammation	(67)
miR-93-5p	Targets ADAMTS9	Inhibits chondrocyte inflammation, autophagy, and apoptosis	(70)

In the human knee joint, resident cells within the infrapatellar fat pad (IPFP) and synovial tissue—such as synovial cells and macrophages—play key roles in the onset and progression of inflammatory joint diseases. Mesenchymal stem cells derived from the IPFP (IPFP-MSCs) possess strong immunomodulatory properties, primarily mediated through their exosomes (MSC^IPFP^-Exos), which can effectively suppress the pro-inflammatory activation of synovial cells and macrophages, thereby modulating local joint inflammation. Previous studies have shown that MSC^IPFP^-Exos exert cartilage-protective effects largely through their abundant miR-100-5p, which specifically inhibits mTOR expression in chondrocytes, thereby activating autophagy. This mechanism markedly alleviates IL-1β–induced chondrocyte apoptosis, promotes ECM synthesis, and reduces the expression of catabolic enzymes such as MMP-13 and ADAMTS5. In a mouse model of destabilization of the medial meniscus, this treatment effectively delayed articular cartilage degeneration and improved gait abnormalities, demonstrating significant chondroprotective effects (68). A study by Yin et al. further compared the therapeutic efficacy of exosomes derived from subcutaneous adipose tissue (ScAT) and those from IPFP-derived ADSCs in KOA. The results showed that IPFP-derived exosomes (Exos^IPFP^) exhibited superior efficacy in inhibiting ECM degradation in chondrocytes compared with ScAT-derived exosomes (Exos^ScAT^). Subsequent small RNA sequencing revealed significantly higher expression of miR-99b-3p in Exos^IPFP^, and functional validation confirmed that it attenuates KOA progression by selectively suppressing ADAMTS4 expression and enhancing ECM synthesis (69).

### 5.3 Synovial mesenchymal stem cell–derived exosomes (SMSC-Exos)

SMSCs are multipotent cells residing in the synovial tissue of joints, characterized by strong self-renewal capacity and the ability to differentiate into chondrocytes, osteocytes, and other cell types (71). The exosomes secreted by these cells act as key mediators of intercellular communication, carrying various bioactive molecules that transmit repair signals to surrounding damaged cells. Previous studies have shown that SMSC-Exos not only effectively suppress joint inflammation but also significantly promote cartilage regeneration and repair. Moreover, by avoiding the potential risks associated with direct live-cell transplantation, SMSC-Exos present substantial therapeutic potential for the treatment of joint diseases such as OA (72).

An *in vivo* study examining the protective role of miRNAs in a rat model of KOA revealed that Wnt5a and Wnt5b, enriched in SMSC-Exos, promote Yes-associated protein (YAP) activation via stimulation of the non-canonical Wnt signaling pathway. This activation enhances chondrocyte proliferation and migration; however, it also suppresses the expression of the key chondrogenic transcription factor SOX9, resulting in reduced ECM synthesis and limited reparative efficacy (73, 74). Notably, the study further demonstrated that engineering exosomes to overexpress miR-140-5p—a miRNA known for its chondroprotective effects—produces markedly different outcomes. These modified exosomes not only promote proliferation and migration through the Wnt/YAP pathway but also counteract the suppression of ECM synthesis, thereby exhibiting more comprehensive cartilage repair and protective effects in KOA (75). Furthermore, recent studies have shown that combining SMSC-Exos with icariin exerts synergistic promotive effects on cartilage repair via the Wnt/*β*-catenin signaling pathway, further underscoring the significant therapeutic potential of SMSC-Exos in KOA treatment (76). Additionally, evidence indicates that miR-485-3p in SMSC-Exos alleviates IL-1β–induced cartilage degradation by targeting NRP1 to inhibit the PI3 K/Akt pathway. This mechanism enhances the proliferation and migration of osteoarthritic chondrocytes while suppressing apoptosis, inflammation, and ECM degradation, thereby slowing the pathological progression of OA.

Researchers have shown that overexpression of specific miRNAs—such as miR-212-5p, miR-155-5p, and miR-26a-5p, as summarized in Table 4—not only promotes the proliferation and migration of osteoarthritic chondrocytes while inhibiting apoptosis, but also effectively relieves the suppression of ECM synthesis and enhances matrix production and secretion. This approach further strengthens the ability of SMSC-Exos to reduce cartilage inflammation and mitigate tissue damage, thereby markedly improving their therapeutic efficacy in OA.

**Table 4:** Mechanisms of action and therapeutic effects of SMSC-exos in the treatment of OA.

RNA	Mechanism	Effect	Reference
miR-140-5p	Blocks inhibition of SOX9 expression through RalA	Relieves suppression of ECM synthesis while promoting chondrocyte proliferation and migration	(75)
miR-212-5p	Targets ELF3	Inhibits chondrocyte degeneration and inflammation	(77)
miR-155-5p	Targets Runx2	Promotes chondrocyte proliferation and migration, inhibits apoptosis, and enhances ECM secretion	(78)
miR-26a-5p	Targets PTEN	Inhibits chondrocyte apoptosis and inflammation and alleviates cartilage damage	(79)
miR-320c	Targets ADAM19	Inhibits chondrocyte apoptosis and promotes repair of cartilage damage	(80)
miR-130b-3p	Targets LRP12 and inhibits LRP12/AKT/β-catenin axis	Alleviates chondrocyte damage	(81)
miR-485-3p	Targets NRP1 and inhibits PI3 K/Akt signaling pathway	Alleviates cartilage degradation	(82)
miR-129-5p	Inhibits IL-1β by suppressing HMGB1 release	Alleviates inflammation and chondrocyte apoptosis	(83)

### 5.4 Synovial fibroblast–derived exosomes (SF-Exos)

Synovial fibroblasts (SFs) originate from the synovial tissue of the inner joint capsule and are key cellular components of the synovial stroma (84). SF-Exos are released by these fibroblasts and mediate communication between SFs and surrounding cells (e.g., chondrocytes, osteoclasts, and immune cells) (85). By transferring their molecular cargo, SF-Exos regulate physiological and pathological processes in recipient cells (86).

A study that established a rat OA model using ACLT + MMx surgery showed that treatment with miR-214-3p–overexpressing SF-Exos improved chondrocyte inflammation and cartilage degeneration while reducing apoptosis, thereby significantly delaying OA progression (87). Similarly, miR-126-3p—by activating anti-inflammatory pathways—effectively suppresses chondrocyte inflammation and delays cartilage degeneration (88). Another study demonstrated that exosomes derived from IL-1β–treated human synovial fibroblasts markedly upregulated MMP-13 and reduced collagen II levels in normal articular chondrocytes *in vitro*. These exosomes also enhanced the migration and tube formation of human umbilical vein endothelial cells and increased proteoglycan release from cartilage explants. Together, these findings suggest that SF-derived exosomes under IL-1β stimulation may contribute to OA pathogenesis by altering chondrocyte metabolic phenotypes and promoting angiogenesis.

Interestingly, the study by Wang et al. showed that intra-articular injection of exosomes derived from miR-146a–overexpressing fibroblast-like synoviocytes (miR-146a-FLS-Exos) effectively alleviated KOA progression. The core mechanism involves the delivery of miR-146a as a functional signaling molecule to joint tissues, where it targets and suppresses TRAF6 expression, thereby inhibiting overactivation of the key TLR4/TRAF6/NF-*κ*B inflammatory pathway. This dual mechanism simultaneously reduces chondrocyte apoptosis and cartilage matrix degradation while promoting the polarization of synovial macrophages from pro-inflammatory M1 to anti-inflammatory reparative M2 phenotypes, ultimately mitigating synovial inflammation (89). SF-Exos thus exhibit significant therapeutic potential by targeting multiple pathological processes in KOA progression. When further engineered, they can serve as efficient drug delivery vehicles for anti-inflammatory molecules or chondroprotective agents, enabling targeted therapies with high efficacy and low toxicity, as summarized in Table 5.

**Table 5:** Mechanisms of action and therapeutic effects of SF-exos in the treatment of OA.

RNA	Mechanism	Effect	Reference
miR-214-3p	Targets anti-inflammatory pathway	Ameliorates chondrocyte inflammation and cartilage tissue degeneration	(87)
miR-126-3p	Targets anti-inflammatory pathway	Constrains chondrocyte inflammation and cartilage degeneration	(88)
miR-146a	Modulates Toll-like receptor 4/TRAF6/NF-κB signaling pathway	Modulates cartilage degradation and macrophage polarization	(89)

### 5.5 Human umbilical cord mesenchymal stem cell–derived exosomes (hucMSC-Exos)

hucMSCs are mesenchymal stem cells isolated from postnatal umbilical cord tissue, capable of releasing substantial amounts of exosomes following *in vitro* expansion (90). hucMSC-Exos play important roles in protecting chondrocytes, promoting cartilage matrix synthesis, and exerting anti-inflammatory and immunomodulatory effects (91). Compared with exosomes derived from BMSCs or ADSCs, hucMSC-Exos offer greater accessibility, exhibit stronger proliferative capacity and higher exosome secretion activity, and avoid ethical concerns associated with other stem cell sources (91, 92).

Studies have shown that miR-1208 in hucMSC-Exos targets and suppresses METTL3 expression, thereby reducing NLRP3 mRNA methylation levels and inhibiting the release of inflammatory factors. At the same time, it prevents the degradation of COL2A1 and aggrecan while suppressing the expression of ADAMTS3 and MMP13. This process markedly reduces the maturation and secretion of key pro-inflammatory cytokines IL-1β and IL-18, effectively alleviating the low-grade inflammatory state in joints. The attenuation of inflammation further inhibits chondrocyte apoptosis, promotes chondrocyte proliferation and migration, and restores the metabolic balance of the ECM, ultimately slowing OA progression (93). Additionally, high levels of lncRNA H19 in hucMSC-Exos promote chondrocyte migration and ECM secretion while inhibiting apoptosis and senescence. This occurs through exosomal H19 acting as a competing endogenous RNA that binds to miR-29b-3p, thereby upregulating FoxO3 expression in chondrocytes (94). A study by Li et al. further revealed that hucMSC-derived exosomes are enriched with several functionally important miRNAs, including miR-122-5p, miR-148a-3p, miR-486-5p, and miR-100-5p. These exosomes activate the PI3 K/Akt signaling pathway, promoting M2 macrophage polarization, reducing the production of pro-inflammatory cytokines (TNF-α, IL-1, and IL-6), and increasing the expression of the anti-inflammatory cytokine IL-10. Consequently, they effectively suppress joint inflammation and delay the pathological progression of KOA (95). Specific details are presented in Table 6.

**Table 6:** Mechanisms of action and therapeutic effects of hucMSC-exos in the treatment of OA.

RNA	Mechanism	Effect	Reference
miR-199a-3p	Inhibits MAPK4/NF-κB signaling pathway	Inhibits chondrocyte inflammation and apoptosis	(96)
miR-1208	Targets METTL3	Alleviates OA progression	(93)
lncRNA H19	Relieves FoxO3 inhibition in chondrocytes through miR-29b-3p	Promotes chondrocyte migration, enhances ECM synthesis, and inhibits apoptosis and senescence, thus facilitating cartilage repair	(94)
miR-122-5p	Regulates PI3K-Akt signaling pathway, thereby promoting polarization of the M2 macrophage phenotype	Inhibits chondrocyte degradation and modulates inflammatory and immune responses	(95)
miR-148a-3p			
miR-486-5p			
miR-100-5p			

Overall, hucMSC-Exos demonstrate strong potential for the treatment of KOA owing to their ready accessibility, potent bioactivity, and broad differentiation capacity.

### 5.6 Human embryonic mesenchymal stem cell–derived exosomes (heMSC-Exos)

heMSCs are pluripotent stem cells derived from early embryonic mesodermal tissue, characterized by high viability and strong multidirectional differentiation potential (97). Exosomes secreted by heMSCs (heMSC-Exos) possess unique advantages in complex tissue regeneration processes. Studies have shown that heMSC-Exos can alleviate oxidative stress through the Nox4–ROS–Nrf2 axis in models of neurological disease (98).

In a study by Zhang et al., using a rat model, weekly intra-articular injections of heMSC-Exos for 12 consecutive weeks produced significant cartilage-specific regenerative effects. This repair mechanism was closely linked to heMSC-Exos modulating local cellular activity through paracrine signaling, thereby promoting chondrocyte proliferation, differentiation, and ECM synthesis (99). Furthermore, a study by Wang et al. established a mouse OA model via surgical destabilization of the medial meniscus. The intra-articular administration of heMSC-Exos effectively mitigated cartilage destruction and matrix degradation. Mechanistic analysis revealed that heMSC-Exos maintained the chondrocyte phenotype and delayed OA progression by promoting type II collagen synthesis and inhibiting ADAMTS5 expression (100).

heMSCs are regarded as a promising strategy for OA treatment because of their multidirectional differentiation potential and remarkable cartilage repair capacity. However, ethical concerns surrounding the use of embryonic tissue remain a major barrier to the clinical application of their derived exosomes.

### 5.7 Human urine stem cell–derived exosomes (huSC-Exos)

huSCs are stem cells isolated from human urine, first identified by Zhang et al. in 2008 (101). These cells exhibit strong self-renewal ability and multidirectional differentiation potential (102). huSCs hold great promise for applications in cell therapy, tissue engineering, and regenerative medicine because of their wide availability, non-invasive and low-cost collection process, and the potential for autologous use, which minimizes immune rejection and avoids ethical issues. Similarly, huSC-Exos have been shown to modulate immune function by inducing B cell proliferation and IgM antibody secretion (103). In addition, when delivered via injectable hydrogel, huSC-Exos can accelerate bone regeneration (104).

In a study on KOA, Liu et al. found that intra-articular injection of huSC-Exos in OA model rats enhanced chondrocyte proliferation and migration while suppressing apoptosis, although it also reduced ECM secretion. Notably, huSC-Exos overexpressing miR-140-5p not only promoted cartilage regeneration and periosteal remodeling but also increased ECM secretion by targeting VEGFA (105). Furthermore, recent studies have engineered exosomes derived from miR-140–overexpressing human urine stem cells. *In vitro* experiments confirmed that these exosomes help maintain mitochondrial function, while *in vivo* intra-articular injection studies demonstrated that they improve subchondral bone structure and delay OA progression (106). The specific content can be found in Table 7.

**Table 7:** Mechanisms of action and therapeutic effects of huSC-exos in the treatment of OA.

RNA	Mechanism	Effect	Reference
miR-140-5p	Targets VEGFA	Increases ECM secretion and promotes regeneration of both cartilage and subchondral bone	(105)
miR140	Targets CAPN1	Modulates chondrocyte mitophagy	(106)

### 5.8 Human amniotic fluid stem cell–derived exosomes (hafSC-Exos)

Amniotic fluid–derived stem cells were first reported and named by Gosden's team in 1983 (107). These cells possess both embryonic stem cell–like pluripotency and adult stem cell characteristics (108). hAFSC-Exos, the nanoscale vesicles secreted by these cells, are notable for their low immunogenicity and high stability. As key bioactive components in cell-free therapies, they exert potent anti-inflammatory, anti-apoptotic, pro-angiogenic, and tissue-repair effects by delivering various bioactive molecules such as proteins and nucleic acids (109, 110). In a monosodium iodoacetate (MIA)–induced rat KOA model, hAFSC-Exos promoted effective cartilage repair through the delivery of TGF*β*. After 3 weeks of treatment, nearly complete cartilage regeneration was observed, characterized by a smooth surface and hyaline cartilage phenotype. This reparative effect was positively correlated with TGF*β* expression levels, highlighting its critical role in the regeneration process (111).

### 5.9 Dental pulp stem cell–derived exosomes (DPSC-Exos)

DPSCs are adult mesenchymal stem cells derived from tooth-associated tissues, offering key advantages such as easy accessibility, minimal invasiveness, and the absence of ethical concerns (112). DPSC-Exos are nanoscale EVs secreted by DPSCs that mediate various biological processes, including tissue repair and immunomodulation (113). A study by Lin et al. demonstrated that DPSC-Exos enriched with miR-140 exert anti-apoptotic effects by regulating the expression of apoptosis-related proteins, thereby alleviating knee joint symptoms in a rat model of OA (114). Fu et al. further revealed that DPSC-Exos suppress osteoclast differentiation by inhibiting TRPV4, suggesting this pathway as a potential therapeutic target for OA treatment (115). Moreover, Karaturk et al. were the first to demonstrate that under hypoxic conditions, DPSC-Exos exert protective effects on osteoarthritic chondrocytes by suppressing the production of pro-inflammatory cytokines (116). Recent studies also indicate that the combined application of *κ*-carrageenan and DPSC-Exos shows significant therapeutic potential in the treatment of rat KOA (117).

### 5.10 Immune cell–derived exosomes

Exosomes derived from immune cells, particularly macrophages and T cells, play critical roles in the treatment of KOA. Their mechanisms primarily involve regulating inflammation, promoting cartilage repair, protecting chondrocytes, and modulating the immune microenvironment (118, 119).

Macrophages are essential immune cells that play central roles in the initiation and progression of inflammation-related diseases (120). Based on their functional phenotypes, macrophages are categorized into pro-inflammatory M1 and anti-inflammatory M2 types (121). Studies suggest that the diverse biological activities of M2 macrophages may be closely related to the exosomes they secrete (122). A study by Qian et al. demonstrated that miR-26b-5p contained in exosomes secreted by M2 macrophages exerts a protective effect on articular cartilage. Mechanistic investigations revealed that this protection occurs through targeting of TLR3 and COL10A1, while miR-26b-5p also contributes to macrophage polarization and promotes chondrocyte proliferation (27). Other studies have shown that exosomes derived from M2 macrophages can exert protective effects in a rat model of OA by activating the PI3 K/AKT/mTOR signaling pathway (123). Moreover, hypoxic preconditioning has been found to further enhance the therapeutic efficacy of M2 macrophage–derived exosomes in OA. The underlying mechanism involves increased delivery of exosomal miR-124-3p to chondrocytes, which suppresses STAT3 expression at the post-transcriptional level, thereby contributing to cartilage protection and inflammation regulation (124).

The development and progression of KOA are closely associated with various T cell subsets, including T helper (Th) cells and regulatory T (Treg) cells (125, 126). Th1 and Th17 cells are pro-inflammatory subsets that exacerbate inflammatory responses and promote disease progression in KOA (127–129). By contrast, Treg cells possess immunosuppressive functions that help maintain immune tolerance, and the exosomes they secrete inherit this regulatory capacity, potentially exerting protective effects in KOA (128, 130, 131). Studies have shown that Treg cell–derived exosomes can effectively suppress inflammatory responses and reduce tissue damage by limiting immune cell infiltration (132).

### 5.11 Exosomes from other sources

Platelet-derived exosomes (Plt-Exos) have been shown to alleviate IL-1β–induced KOA in human chondrocyte models by promoting chondrocyte proliferation and migration, suppressing inflammatory responses, and improving subchondral bone microstructure. RNA sequencing analysis revealed that the differentially expressed genes following Plt-Exos treatment were significantly enriched in biological processes related to anti-inflammatory responses (e.g., inhibition of the NF-*κ*B signaling pathway), as well as cell adhesion and migration (133). Previous studies have also shown that exosomes derived from induced pluripotent stem cells (iPSC-Exos) contribute to mitigating ECM degradation and the pathogenesis of OA (134). In a rabbit ACLT-induced OA model, Hsueh et al. observed that intra-articular injection of iPSC-Exos reduced cartilage degeneration, as evidenced by decreased structural damage, upregulation of collagen II expression, and downregulation of MMP13, ADAMTS5, and TNF-α (135). Furthermore, a study by Zhu et al. demonstrated that in a mouse OA model, exosomes derived from induced pluripotent stem cell–derived mesenchymal stem cells (iMSC-Exos) exhibited superior therapeutic efficacy compared to those secreted by synovial membrane mesenchymal stem cells (SM-MSC-Exos) (134).

## 6 Potential of engineered exosomes in treatment of KOA

Although exosomes have opened new avenues for the treatment of KOA, the clinical application of natural exosomes remains limited by challenges such as suboptimal therapeutic efficiency, insufficient targeting precision, and short duration of action (136). By contrast, engineered exosomes can act synergistically at multiple levels to precisely modulate the complex pathological processes of KOA, showing significant potential to address its core disease mechanisms (137). To improve *in vivo* retention and therapeutic efficacy, one study developed injectable hyperbranched PEG–crosslinked hyaluronic acid hydrogel microparticles using microfluidic technology to encapsulate miR-99b-3p–modified exosomes. This approach significantly prolonged intra-articular retention of exosomes, enhanced their long-term therapeutic effects in promoting cartilage repair and inhibiting ECM degradation, and provided a clinically translatable strategy for sustained KOA treatment (69). The Wan team designed engineered exosomes modified with targeting peptides and encapsulated within a photocrosslinked spherical hydrogel. Both *in vitro* and *in vivo* studies confirmed that this composite hydrogel effectively loaded LRRK2-IN-1 and markedly enhanced the targeting ability and intra-articular retention of exosomes. This system successfully preserved the intrinsic functions of exosomes in alleviating OA and promoting cartilage repair while overcoming limitations such as rapid clearance and low retention rate, offering a potential new nanotechnology-based strategy for the treatment of OA (138). Furthermore, mechanistic investigations revealed that TNF-α preconditioning activates the PI3 K/AKT signaling pathway in IPFP-MSCs, upregulates expression of the autophagy-related protein ATG16L1, and consequently promotes exosome secretion. In a mouse OA model, intra-articular injection of the resulting IPFP-MSC-Exos^TNF−*α*^ demonstrated superior efficacy in ameliorating joint pathology compared with conventional exosomes. Subsequent research found that this enhanced chondroprotective effect was associated with a marked increase in low-density lipoprotein receptor–related protein 1 (LRP1) content within the exosomes. Together, these findings highlight an effective strategy to improve both production yield and therapeutic efficacy for OA (138).

## 7 Exosome challenges in knee osteoarthritis treatment

The application of exosomes in the treatment of KOA still faces several critical challenges. First, standardized protocols for exosome preparation have yet to be established. Different isolation and purification techniques—such as ultracentrifugation, polymer-based precipitation, and size-exclusion chromatography—yield substantial differences in purity, concentration, and biological activity, resulting in difficulties with quality control and comparability across exosomes from different sources or batches (139, 140). Second, there is no consensus on dosage or administration regimens for exosome-based therapy. Determining a safe, effective, and optimal dosage for patients with KOA at different disease stages remains a major challenge and requires further systematic preclinical and clinical studies (141–144). Another significant obstacle involves exosome stability during storage. Exosomes are prone to degradation after repeated freeze–thaw cycles, which can compromise their structural integrity and reduce bioactive content. They may also undergo denaturation or inactivation under both excessively low and high temperatures (145). Thus, even after successful isolation, exosomes must be stored within an appropriate temperature range for short-term preservation to maintain their structural and functional stability. Likewise, suitable strategies for long-term storage are still needed (146, 147). Finally, the long-term safety and mechanisms of action of exosome-based therapies remain insufficiently understood. The scarcity of comprehensive animal studies and clinical data presents a major barrier to confirming their long-term efficacy and biosafety. Collectively, these challenges continue to hinder the reliable clinical translation of exosome therapies for KOA (148, 149).

## 8 Discussion

**Figure 3:**
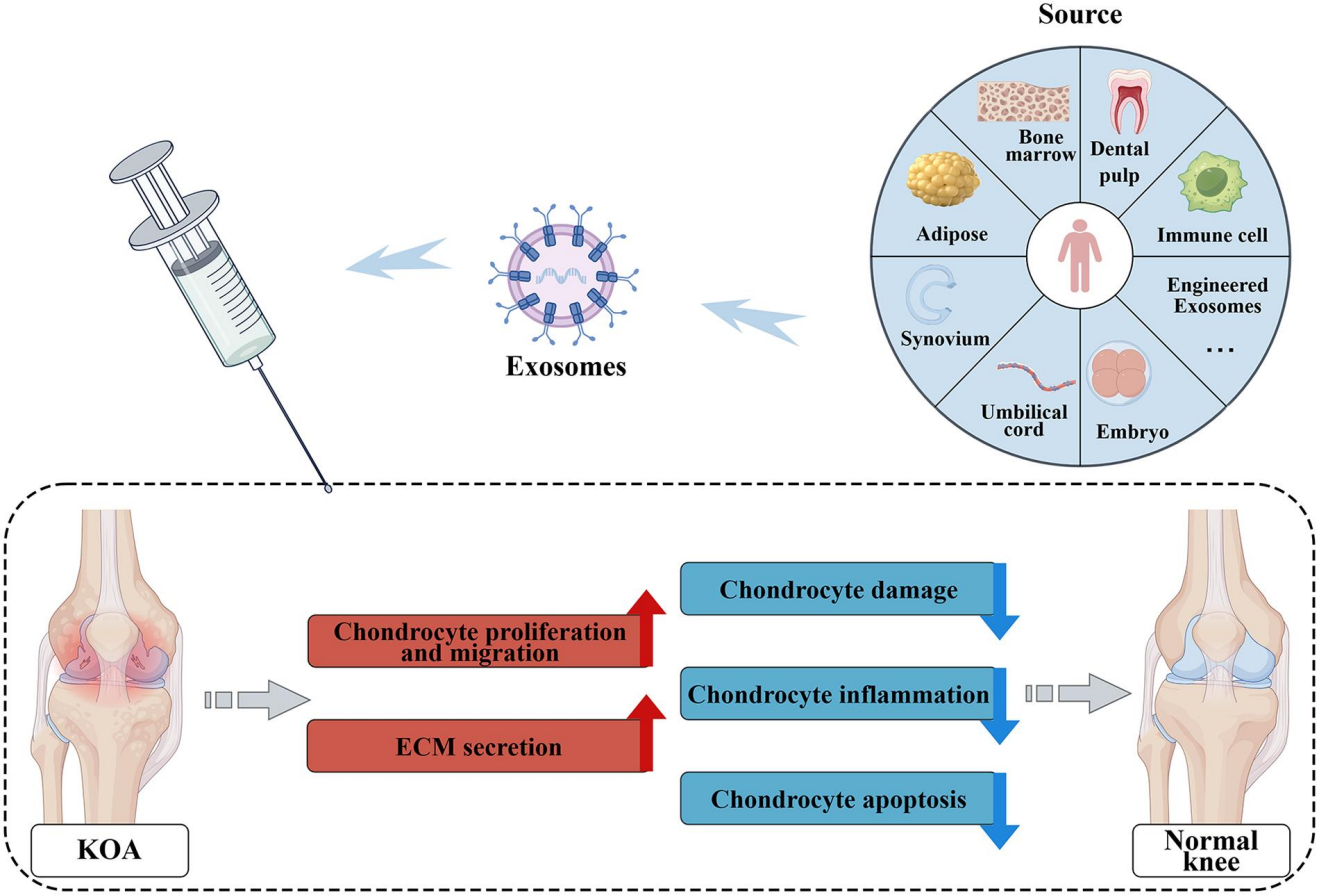
Application of exosomes from diverse sources in KOA.

Exosomes deliver bioactive components such as proteins and RNAs, providing important therapeutic strategies for KOA (150). They act synergistically to exert multiple effects—including anti-inflammatory activity, cartilage regeneration, regulation of matrix metabolism, and antioxidant protection—thereby promoting remodeling of the joint microenvironment and facilitating cartilage repair (Figure 3) (151).Among the various exosome types studied, engineered exosomes in particular demonstrate enhanced targeting precision and therapeutic efficacy, highlighting their promising potential for future KOA treatment (152).

Exosomes exhibit significant variation depending on their cellular origin in Table 8. The ease of obtaining these exosomes differs considerably. Sources such as human urine-derived stem cells, platelets, and tissues like adipose and umbilical cord are relatively accessible. In contrast, acquiring exosomes from bone marrow, synovium, or embryonic sources typically requires invasive procedures or more complex isolation methods. Production yield also varies widely. Exosomes from adipose tissue, umbilical cord mesenchymal stem cells, and platelets generally offer a higher potential yield. Non-invasive sources like urine often depend on *in vitro* cell expansion, which can limit the final quantity of exosomes obtained (153). Ethical considerations present another layer of complexity. Sources like umbilical cord, dental pulp, and processed medical waste are associated with fewer ethical concerns. The use of embryonic stem cells, however, faces strict regulations due to the involvement of embryos (154). Regarding clinical translation, most exosome-based therapies remain in preclinical or early-stage clinical research. No source has yet been approved for routine clinical treatment. Functionally, exosomes from these diverse origins commonly demonstrate therapeutic potential in areas such as tissue repair, immune modulation, and disease modeling. Certain sources, including immune cells and platelets, also show promise for developing diagnostic biomarkers (118, 155). In summary, selecting an exosome source requires a balanced consideration of multiple factors. These include acquisition feasibility, ethical acceptability, scalability, and biological relevance to the target disease. Future progress will depend on standardizing preparation methods and obtaining robust clinical validation to enable their transition into practical applications.

**Table 8:** The differences of exosomes from from diverse cellular sources.

Source	Collection method	Output (relative comparison)	Ethical considerations	Research stage	Current Status (Clinical Use)	Functionality relevance
BMSC	Invasive (bone marrow puncture)	Medium	Low	Preclinical	Not widely approved	Therapeutic
ADSC	Minimally invasive (liposuction)	High	Low	Preclinical	Not widely approved	Therapeutic
SMSC	Invasive (obtained through arthroscopy or surgery)	Medium	Low	Preclinical	Unapproved	Therapeutic
SF	Invasive (obtained through arthroscopy or surgery)	Medium	Low	Preclinical	Unapproved	Therapeutic
hucMSC	Non-invasive (disposal of umbilical cord after delivery)	High	Low	Early clinical trials	Not widely approved	Therapeutic
heMSC	Invasive and complex	Low	High	Preclinical	Unapproved (Strictly limit)	Therapeutic
huSC	Non-invasive	Low	Low	Preclinical	Unapproved	Therapeutic
hafSC	Invasive (amniocentesis)	Medium	Medium	Preclinical	Unapproved	Therapeutic
DPSC	Minimally invasive (obtained after tooth extraction)	Medium	Low	Preclinical	Unapproved	Therapeutic
Immune cell	Invasive (separation of blood or tissue)	Low	Medium	Preclinical	Unapproved	Therapeutic and diagnostic
blood platelet	Minimally invasive (blood collection)	High	Low	Preclinical	Unapproved	Therapeutic
iPSC	Non-invasive (somatic cell reprogramming)	Dependent cultivation system	Medium	Preclinical	Unapproved	Therapeutic

Although exosome-based therapy still faces numerous challenges, continued interdisciplinary collaboration is expected to foster innovative strategies that enhance its therapeutic effectiveness for OA. The combination of exosomes with other treatment modalities holds considerable promise for KOA. When used alongside conventional drugs, exosomes can not only enhance drug efficacy and reduce side effects but also serve as targeted delivery vehicles that precisely transport therapeutic agents to affected joint regions (156). This approach increases local drug concentration, minimizes systemic exposure, and reduces the risk of systemic adverse reactions. Moreover, the intrinsic immunomodulatory and tissue-repair functions of exosomes can act synergistically with pharmacologic treatments to jointly alleviate inflammation and promote cartilage repair, thereby integrating and amplifying multiple therapeutic mechanisms (157, 158). Future research should focus on improving the sensitivity, specificity, and therapeutic efficacy of exosomes. As key mediators of intercellular communication, exosomes carry abundant biological information. By further elucidating the complex cellular interactions and signaling networks involved in OA progression, researchers may uncover new therapeutic targets and develop more precise exosome-based interventions.

Compared to prior reviews, this article offers a more systematic overview of recent advances in exosome-based diagnosis and treatment for osteoarthritis. A key focus lies in the therapeutic domain, where it summarizes the mechanisms and research trends of exosomes derived from different stem cell sources. For clarity, the distinct therapeutic mechanisms and effects of various exosomes are presented in a comparative table. The article further details specific action pathways of exosomes in knee osteoarthritis. It also introduces emerging strategies for engineering exosomes, highlighting how bioengineering can enhance their therapeutic efficacy. Finally, this work objectively outlines the major current challenges in exosome therapy and suggests potential directions for future research.

This article has several inherent limitations. First, as a narrative review, the selection of cited literature is inherently subjective. It does not adhere to the rigorous search and screening protocols characteristic of a systematic review. Although we endeavored to cover significant advancements, the final selection of articles was inevitably influenced by the authors' perspectives and research focus. Second, this review did not include a formal methodological quality assessment of the cited studies. Consequently, studies with potential design flaws or biased conclusions may have been discussed alongside more robust research. This approach could affect the overall reliability of the integrative findings. Finally, the primary aim of this article is to synthesize existing knowledge and to propose insights and future directions, rather than to deliver definitive conclusions. The mechanisms and perspectives discussed are largely based on inferences and interpretations drawn from the literature. They are intended to inspire subsequent research. Their broader applicability and validity require future confirmation through rigorous basic science and clinical investigations.

## 9 Conclusion

In summary, as key mediators of intercellular communication, exosomes show great promise in the diagnosis and treatment of KOA. Exosomes derived from various sources—such as BMSCs, ADSCs, and immune cells—offer diverse therapeutic applications, including modulation of the inflammatory microenvironment, promotion of cartilage regeneration, and potential use as non-invasive biomarkers for early diagnosis. However, several challenges remain, including uncertainty regarding the optimal exosome type and administration frequency, difficulties in large-scale production, risks of off-target effects during delivery, and incomplete understanding of their underlying mechanisms of action. Engineered exosomes, through targeted modifications, can enhance delivery precision and therapeutic efficacy, providing new opportunities for standardized manufacturing and combination therapies. Looking ahead, the application of exosomes in KOA treatment holds broad potential and may contribute to the advancement of personalized medicine. Nevertheless, there is an urgent need to optimize isolation and purification techniques, improve tissue-specific targeting, develop efficient delivery systems, and validate safety and efficacy through large-scale clinical trials to enable successful clinical translation.
